# Mapping the Influence of the Gut Microbiota on Small Molecules across the Microbiome Gut Brain Axis

**DOI:** 10.1021/jasms.1c00298

**Published:** 2022-03-09

**Authors:** Heather Hulme, Lynsey M. Meikle, Nicole Strittmatter, John Swales, Gregory Hamm, Sheila L. Brown, Simon Milling, Andrew S. MacDonald, Richard J.A. Goodwin, Richard Burchmore, Daniel M. Wall

**Affiliations:** † Institute of Infection, Immunity and Inflammation, College of Medical, Veterinary and Life Sciences, Sir Graeme Davies Building, University of Glasgow, Glasgow G12 8TA, United Kingdom; ‡ Imaging and Data Analytics, Clinical Pharmacology and Safety Sciences, Biopharmaceuticals R&D, AstraZeneca, Cambridge CB4 0WG, U.K.; § Lydia Becker Institute of Immunology and Inflammation, Faculty of Biology, Medicine and Health, Manchester Academic Health Science Centre, University of Manchester, Manchester M13 9NT, U.K.

**Keywords:** mass spectrometry imaging, microbiome, brain, neurotransmitters, metabolites

## Abstract

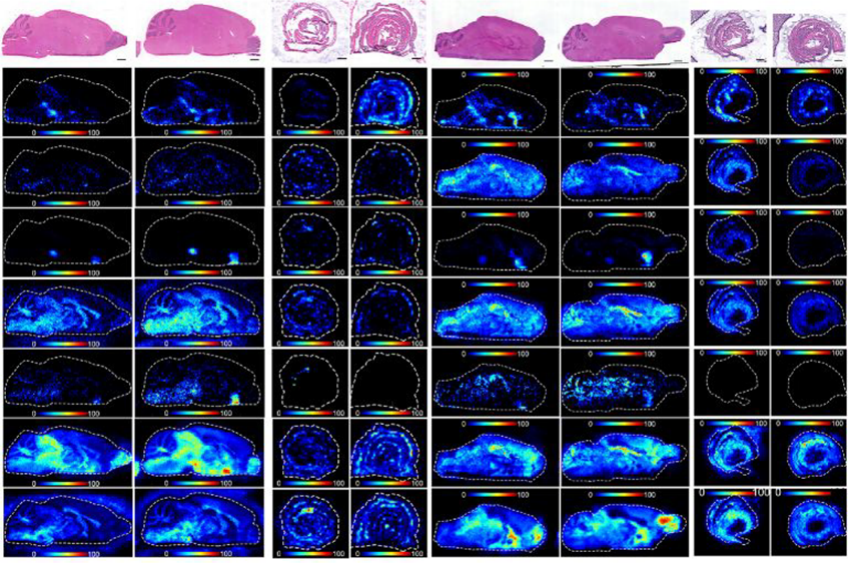

Microbes exert influence across the microbiome–gut–brain axis through neurotransmitter production, induction of host immunomodulators, or the release or induction of other microbial or host molecules. Here, we used mass spectrometry imaging (MSI), a label-free imaging tool, to map molecular changes in the gut and brain in germ-free, antibiotic-treated and control mice. We determined spatial distribution and relative quantification of neurotransmitters and their precursors in response to the microbiome. Using untargeted MSI, we detected a significant change in the levels of four identified small molecules in the brains of germ-free animals compared to controls. However, antibiotic treatment induced no significant changes in these same metabolites in the brain after 1 week of treatment. This work exemplifies the utility of MSI as a tool for the study of known and discovery of novel, mediators of microbiome-gut-brain axis communication.

## Introduction

Deciphering the complex bidirectional communication across the microbiome gut brain (MGB) axis remains a challenging prospect. The composition and stability of the gut microbiome is now proposed to be a significant contributor to human health with changes in its composition suggested as a contributing factor in a number of neurological conditions. Diverse human neurological disorders, ranging from autism spectrum disorders (ASDs) and attention-deficit/hyperactivity disorder (ADHD) to Alzheimer’s and Parkinson’s disease, have linked gastrointestinal abnormalities or changes in the gut microbiome.^1−6^ Similarly, altered levels of certain bacterial species in the gut have been linked with depression.^7,8^

Despite the deficiencies of germ-free (GF) animal models, their use for investigating links between the gut microbiota and the brain have proved informative and are helping to uncover the influence of bacteria on neurotransmitter levels alongside other bacterial and host metabolites. Their neuro-developmental abnormalities including increased blood–brain–barrier (BBB) permeability, alterations in abundance and maturity of microglia cells, and reduced myelination^9−11^ have been well documented, but their use, alongside antibiotic-treated (ABX) mice, has provided further evidence that supports the importance of a stable and healthy gut microbiota in maintaining normal cognitive function and development.^12^ GF mice display reduced anxiety-like behavior and an increased response to stress that is fully alleviated upon colonization with *Bifidobacterium infantis*,^12−15^ while murine ABX treatment models reinforce the significance of the MGB axis with treated mice showing impaired cognition and significantly altered behaviors that can be linked to the absence of particular bacteria.^16−19^ These effects are likely mediated by the numerous bacterial molecules including short chain fatty acids (SCFAs), phenolic acids, quorum sensing peptides, peptidoglycan, and lactic acid, that have been proposed to exert influence across the MGB axis.^20−22^ SCFAs both cross and modulate the permeability of the BBB, while animals and humans are also dependent on intestinal microbes to produce or supplement their vitamin needs.^10,23,24^

Signaling across the MGB axis can occur via the vagus nerve, bacterial modulation of host immune responses or host neurotransmitter production, and signaling through bacterial neurotransmitters or other microbial molecules. Microbes produce a number of neurotransmitters including γ aminobutyric acid (GABA), norepinephrine and dopamine, serotonin, and acetylcholine, while bacterial production or depletion of essential neurotransmitter intermediates such as tryptophan can also affect host production.^14,19,25−30^ Manipulation of the microbiome can also alter host neurotransmitter levels as microbes stimulate their intestinal production, with depletion of the microbiome or its supplementation with probiotic bacteria, capable of altering specific levels of neurotransmitters even in the brain.^31−35^

Understanding how the gut microbiota influence the brain, and the complex network of molecules and neurotransmitters that mediate this influence, is a significant challenge requiring novel tools and approaches. Mass spectrometry imaging (MSI) is a molecular imaging tool that can be applied to understand and map biological systems with recent successes in mapping microbial interactions with their environment.^36−38^ MSI maps the distribution of small molecules across a tissue section independent of any label, so no prior knowledge of the molecules present is required. The ability to detect spatial distribution and abundance of thousands of compounds simultaneously in tissue sections makes this a powerful approach for studying the MGB axis. Here, using MSI, we detected significant differences in neurotransmitter levels and those of their precursors in the guts of both GF and ABX mice. However, in the brain only tryptophan levels were significantly affected in ABX-treated mice compared to controls, with significant changes detected in every brain region imaged. Through untargeted MSI we also discovered four small molecules that were significantly changed in the GF brain. We focused on two of these, identified as 3-hydroxy-3-methylglutaric acid (3-HMG) and pantothenic acid (vitamin B5). 3-HMG, a metabolite associated with oxidative stress, was significantly increased in the GF brain, while vitamin B5, which can be produced by the microbiome, was present at a lower level in GF brain and is implicated in brain health. This work indicates that the brain remains largely protected from microbiome changes in the gut and indicates plasticity, not just in the brain, but across the MGB axis in GF mice.

## Methods

### Animal Models Used in This Study

#### Germ-Free Studies

All germ-free (GF) work was undertaken at the University of Manchester Gnotobiotic Facility. Five GF and specific pathogen free (SPF) mice were used in this study, which were male mice, aged 7–8 weeks, on a C57BL/6J background strain. Both experimental groups were fed the same pelleted diet that was irradiated with 50 kGy to ensure sterility. The Manchester Gnotobiotic Facility was established with the support of the Wellcome Trust (097820/Z/11/B), using founder mice obtained from the Clean Mouse Facility (CMF), University of Bern, Bern, Switzerland.

#### Antibiotic Studies

All work involving antibiotic (ABX) treatment was undertaken at the University of Glasgow. Five ABX-treated animals and corresponding untreated controls were used in these studies and were male mice, aged 7–8 weeks, on a C57BL/6J background strain. The ABX cocktail consisted of 1 mg/mL gentamicin, 1 mg/mL neomycin, and 0.5 mg/mL vancomycin in sterile distilled drinking water. ABX supplemented drinking water was provided ad libitum for a period of 1 week and refreshed every 2 days. The untreated controls were given sterile drinking water without ABXs ad libitum, which again was refreshed every 2 days. Both treated and control groups were fed the same standard chow. All antibiotics selected for this study were chosen based on their specificity for the gut microbiota; to the best of our knowledge, the antibiotics used are not absorbed by the intestine. Approval for these procedures was given prior to their initiation by internal University of Manchester and University of Glasgow ethics committees and by the U.K. Home Office under licenses 70/7815, PPL40/4500, P64BCA712, and P78DD6240.

### Tissue Processing

Mice were culled by cervical dislocation, and brains and colons were removed. Brains were placed in a mold and were immediately frozen using a slurry of dry ice and ethanol to maintain structural integrity and to ensure that all biochemical processes were halted. The colon samples were then cut lengthwise over ice, the fecal matter was removed, and the remaining GI tissue was then rolled using the “Swiss roll technique” before embedding in 2.5% medium viscosity carboxymethyl cellulose (Sigma-Aldrich, Dorset, UK). Both brains (in sagittal orientation) and colons were sectioned using a CM3050S cryostat microtome (Leica Biosystems, Nussloch, Germany) to 10 μm thickness at −18 °C. Colon and brain sections were thaw mounted onto either indium tin oxide (ITO) coated slides for matrix assisted laser desorption/ionization (MALDI)-MSI or normal microscope slides for desorption electrospray ionization (DESI)-MSI. Tissue sections from the different conditions were randomly located on each slide and the slides run in different orientations. Each slide contained sections from both conditions that were being compared; for example, both GF and SPF sections were mounted on the same slide and imaged in the same mass spectrometer experiment. This was to ensure the sections from the different conditions were prepared identically and to control for any possibility of artifacts occurring during derivatization agent spraying or due to variation in mass spectrometer signal across the slide. For the brain, stereotactically matched sections were selected to ensure that all comparisons were performed using corresponding brain regions. Consecutive sections to those for MSI were collected for histology purposes. Slides were prepared and stored at −80 °C until required for analysis. Prior to derivatization, matrix application, or analysis, the slide was taken from −80 °C and brought to room temperature under a stream of air.

### Neurotransmitter Derivatization

The derivatization of primary amine neurotransmitters was performed as previously described.^39^ Nine milligrams of 2,4-diphenylpyranylium tetrafluoroborate (DPP-TFB) was added to 1.2 mL of 100% methanol and sonicated for 20 min (min). This was then gradually added to 6 mL of 70% methanol in water with 3.5 μL of trimethylamine. This solution was sprayed onto the tissue for derivatization using an automated sprayer (TM-Sprayer, HTX Technologies); 30 passes were performed using a nozzle temperature of 75 °C, velocity of 1100 mm/min, flow rate of 80 μL/min, and gas pressure of 6 psi. After coating, the slide was incubated in a Petri dish with vapor from a 50% methanol/water solution three times for 5 min each.

### DESI-MSI Analysis

DESI-MSI was performed on a Q-Exactive mass spectrometer (Thermo Scientific, Waltham, MA) equipped with an automated 2D DESI source (Prosolia, Inc., Indianapolis, IN). A home-built DESI sprayer assembly, as described previously,^40^ was used with the spray tip positioned at 1.5 mm above the sample surface and at an angle of 75°. The distance between the sprayer to mass spectrometer inlet was 7 mm with a collection angle of 10° and <1 mm distance between inlet and sample surface. The spray solvent was methanol/water (95:5 v/v) delivered at 1.5 μL/min using a Dionex Ultimate 3000 pump (Thermo Scientific, Waltham, MA) at a spray voltage of ±4.5 kV. Nitrogen was used as the nebulization gas at a pressure of 7 bar. General instrument settings used to image specific molecules are shown in Table 1. For acquisition of MS/MS spectra, an injection time of 300 ms, mass resolution of 70000, and a mass isolation window of ±0.3 Da was used. For MS/MS imaging of metabolites with mass to charge (*m*/*z*) ratios 161.044 and 218.103, various fragmentation higher collision induced dissociation (HCD) settings were used at a spatial resolution of 100 μm.

**Table 1:** DESI-MSI Parameters

molecule	pump solvent delivery (μL/min)	spatial resolution (μm)	ionization mode	mass range (*m/z)*	S-lens setting (V)	mass resolution	injection time (ms)
neurotransmitters	1.5	100	positive	200–800	75	35000	150
pantothenic acid	1.5	100	negative	65–400	50	70000	300
3-hydroxy-3-methylglutaric acid	1.5	100	negative	65–400	50	70000	300

Data was recorded as individual line scans and converted into imzML format using imzML converter version 1.1.4.5^41^ and visualized using MSiReader version 0.09.^42^ Imaging data from analysis of the brains was normalized by total ion count due to ion suppression effects in different areas of the brain; data from analysis of colons was not normalized. First-order linear interpolation was used for image generation. All mean intensities of the molecules of interest were determined across the entire tissue section or brain region analyzed. Brains were divided into regions for relative quantitation of neurotransmitters and metabolites. Major regions were annotated according to the Allen sagittal mouse brain atlas; the cortex (cor), hippocampus (hipp), hypothalamus (hyp), thalamus (thal), striatum (stri), midbrain (mid), pons, medulla (med), white matter of the cerebellum (Cb white), gray matter of the cerebellum (Cb gray), and corpus callosum (cc).^43,44^ The substantia nigra (SN) was also imaged due to the high abundance of neurotransmitters such as dopamine and serotonin found in this area. An annotated H&E stain of a brain depicting the brain regions is shown in Supplementary Figure 1. The mean intensity was used to normalize by the area of the region analyzed, which controls for any difference in size of the tissue sections or regions between mice. Relative quantitation was performed on the most abundant peak in the mass spectrum for each metabolite. For all derivatized neurotransmitters and the metabolite at *m*/*z* 161.044, only the [M + H]^+^ ion was detected. For the metabolite at *m*/*z* 218.103, both the [M – H]^−^ and [M + Cl]^−^ ions were detected; however, the [M + Cl]^−^ was very low in abundance and not detected in some brain regions; therefore, the [M – H]^−^ was used for relative quantitation as it would be more accurate (Supplementary Figure 2). The data from the colons was transformed to log10 prior to statistical analysis, and the relative abundance values for the brain were not transformed. A Shapiro–Wilk normality test was performed on the data to check for a normal distribution. If the data passed the normality test an unpaired *t* test was performed. If the data failed the test, a Mann–Whitney U test was performed. When biological replicates were analyzed over two separate DESI-MSI experiments a paired *t* test was performed instead.

### H&E Staining of Brains and Colons

Brain and colon sections that had undergone MSI analysis were H&E stained postimaging to permit localization of candidate metabolites and neurotransmitters to specific brain regions. Sections were fixed on the slide in ice-cold 75% acetone and 25% ethanol for 10 min and air-dried for a further 10 min. Slides were placed in water for 2 min, submerged in hematoxylin (Sigma-Aldrich, Poole, Dorset, UK) for 2 min, and immediately rinsed in cold running water. The slides were then dipped for 3 s in acid alcohol 0.5% (Atom Scientific Hyde, Cheshire, UK) and rinsed in water before submerging in Scott’s tap water (Atom Scientific Hyde, Cheshire, UK) for a further 30 s. The sections were counter-stained with eosin (Sigma-Aldrich, Poole, Dorset, UK) for 2 min and washed in water. Sections were then dehydrated in increasing concentrations of ethanol (70% ethanol for 30 s, 90% for 1 min, and twice for 3 min in 100% ethanol), cleared in xylene (twice for 3 min), and coverslipped using DPX mounting media (Atom Scientific, Hyde, Cheshire, UK).

## Results

### Targeted Neurotransmitters Remain Unaffected by the Lack of a Gut Microbiota

**Figure 1:**
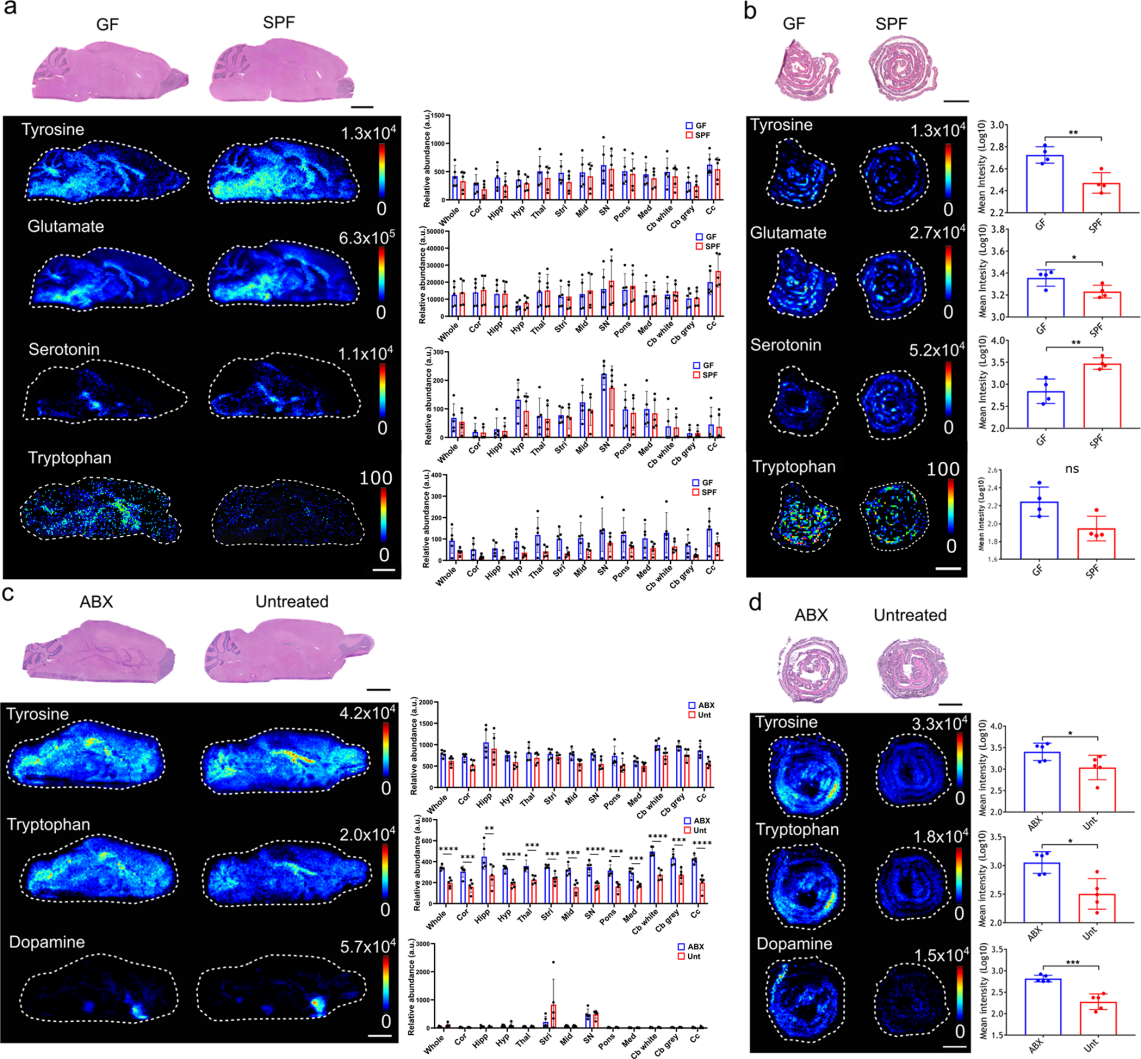
Effects of the gut microbiome on neurotransmitters, neurotransmitter precursors, and neurotransmitter metabolites in the murine brain and gut. DESI-MS images of various DPP-derivatized neurotransmitters, neurotransmitter precursors, or neurotransmitter metabolites in (a) germ-free (GF) and specific-pathogen-free (SPF) mouse brains (*N* = 5), (b) GF and SPF mice colons (*N* = 4), (c) antibiotic treated (ABX) and untreated (Unt) mouse brains (*N* = 5), and (d) ABX and Unt mice colons (*N* = 5). H&E stained sections are shown, which are the same tissue sections that underwent DESI-MSI analysis. Tyrosine ([M + H]^+^*m*/*z* 396.159), tryptophan ([M + H]^+^*m*/*z* 419.175), dopamine ([M + H]^+^*m*/*z* 368.165), glutamate ([M + H]^+^*m*/*z* 362.139), and serotonin ([M + H]^+^*m*/*z* 391.181). The bar plots show the average metabolite abundance (ion intensity) across the whole tissue sections in the different groups tested. The bar plots for the brain also show relative abundance of neurotransmitters in multiple brain regions the cortex (cor), hippocampus (hipp), hypothalamus (hyp), thalamus (thal), striatum (stri), midbrain (mid), pons, medulla (med), white matter of the cerebellum (Cb white), gray matter of the cerebellum (Cb gray), the corpus callosum (cc) and the substantia nigra (SN). For (b), statistical analysis was performed using an unpaired *t* test. For (c) and (d), statistical analysis was performed using a paired *t* test, an unpaired *t* test or a Mann–Whitney U test where appropriate. For individual brain regions statistical analysis was performed using an unpaired *t* test. The *p* values were corrected for multiple testing by False Discovery Rate (FDR), two-stage step-up method. The desired FDR was set to 1%. The asterisks on the bar plot show significance with a *p* value of 0.01 (**), 0.001 (***), or ≤0.001 (****). Brain regions without asterisks were not significantly different in levels of neurotransmitter or metabolite. Error bars represent standard deviation. Scale bars = 2 mm. Ion images show target *m*/*z* ± 0.005 Da.

The brains and colons from germ-free (GF) and conventionally colonized, specific-pathogen-free (SPF) control mice were first compared by MSI using a targeted approach. DPP-derivatization of primary amines was performed to allow targeted imaging of neurotransmitters, neurotransmitter precursors, and neurotransmitter metabolites: serotonin, tryptophan, dopamine, tyrosine, 3-methoxytyramine, GABA, and glutamate (Figure 1a,b).^39,45,46^ Significant differences in the levels of several neurotransmitters were observed in the gut. Serotonin was lower (3.8-fold decrease) in the GF mouse gut (Figure 1b; *P* ≤ 0.01), while glutamate (1.3-fold increase) (Figure 1b; *P* ≤ 0.05) and the dopamine precursor tyrosine (1.8-fold increase) (Figure 1b; *P* ≤ 0.01) were significantly increased in the GF gut. There were no differences detected in the levels of tryptophan, dopamine, 3-methoxytyramine, or GABA in the gut of GF mice (Supplementary Figure 3). Changes in serotonin, glutamate, and tyrosine levels in the intestine were not reflected in the corresponding brain sections of GF mice where no significant differences were identified for any of the seven neurotransmitters or their precursors across whole brain sections (Figure 1a). Targeted MSI of specific brain regions (cortex, hippocampus, hypothalamus, thalamus, striatum, midbrain, substantia nigra, pons, medulla, white matter cerebellum, gray matter cerebellum, corpus callosum) was undertaken to further investigate these findings further, but no significant change was seen in the targeted neurotransmitters or precursors in any region of the GF mouse brain when compared to the corresponding region in control mice (Figure 1a).

### MSI of Neurotransmitters in the Brains and Colons of ABX-Treated Mice

To determine whether an acute disruption of the microbiota over 7 days could have an influence on the levels or the localization of the same neurotransmitters in the brain, the colon and the brains of ABX-treated mice were imaged in a targeted manner.

Tyrosine levels were changed in ABX-treated mice in a manner similar to that seen in GF mice with a significant increase in the colon (2.2-fold), but again, this intestinal increase in tyrosine levels was not reflected in levels of tyrosine across whole brain sections (Figure 1c,d). Unlike GF mice, ABX-treated mice showed no significant change in the levels of serotonin in either the colon or brain compared to untreated mice (Supplementary Figure 4). However, there was a significantly higher abundance of tryptophan in both the colon (3.2-fold; *P* ≤ 0.05) and brain (1.7-fold; *P* ≤ 0.01) of ABX-treated mice compared to controls (Figure 1c,d). Imaging of individual brain regions indicated that tryptophan levels were significantly increased in each individual region in the ABX mice in comparison to control mice, with the highest fold changes observed in the corpus callosum (2.1-fold), the midbrain (2.1-fold), and the substantia nigra (2-fold). Dopamine levels were also significantly higher (3.3-fold; *P* ≤ 0.001) in the colons from ABX-treated mice compared to untreated, but no significant change was detected across the whole brain or in the striatum or substantia nigra, where dopamine was most abundant (Figure 1d). No significant difference was detected in levels of 3-methoxytyramine, glutamate or GABA in either the gut or brain sections imaged (Supplementary Figure 4).

### Untargeted MSI to Detect Novel Molecular Changes in GF Mouse Brains

As no significant changes were seen in several neurotransmitters in GF brains, untargeted imaging was performed using DESI-MSI on the colon and brains of GF mice to probe the MGB axis for molecular changes induced by microbiota disruption. Full scan spectra were collected from *m*/*z* 65–400 and 250–1000 in both positive and negative ionization mode, allowing detection of a wide range of metabolites and not targeted toward a particular group. Significant differences were detected in only three masses when comparing GF and SPF mouse brains. Two of these identified metabolites at *m*/*z* of 218.1030 and 161.0446, both detected in negative-ion mode, were selected for further analysis as putative identities could be assigned from online databases, as discussed further below. The third mass at *m*/*z* 160.133 was below the limit of detection in GF brains and could not be assigned an identity from online databases. The identity of this mass as two microbiome-derived metabolites of identical elemental composition, 3-methyl-4(trimethylammonia)butanoate and 4-(trimethylammonio)pentanoate), was finally determined via two-dimensional NMR after isolating the producing bacterial species, with the associated structures and their bacterial origin recently described.^47^

### Levels of 3-Hydroxy-3-methylglutaric Acid (3-HMG, *m*/*z* 161.0446) Are Increased in the GF Brain

**Figure 2:**
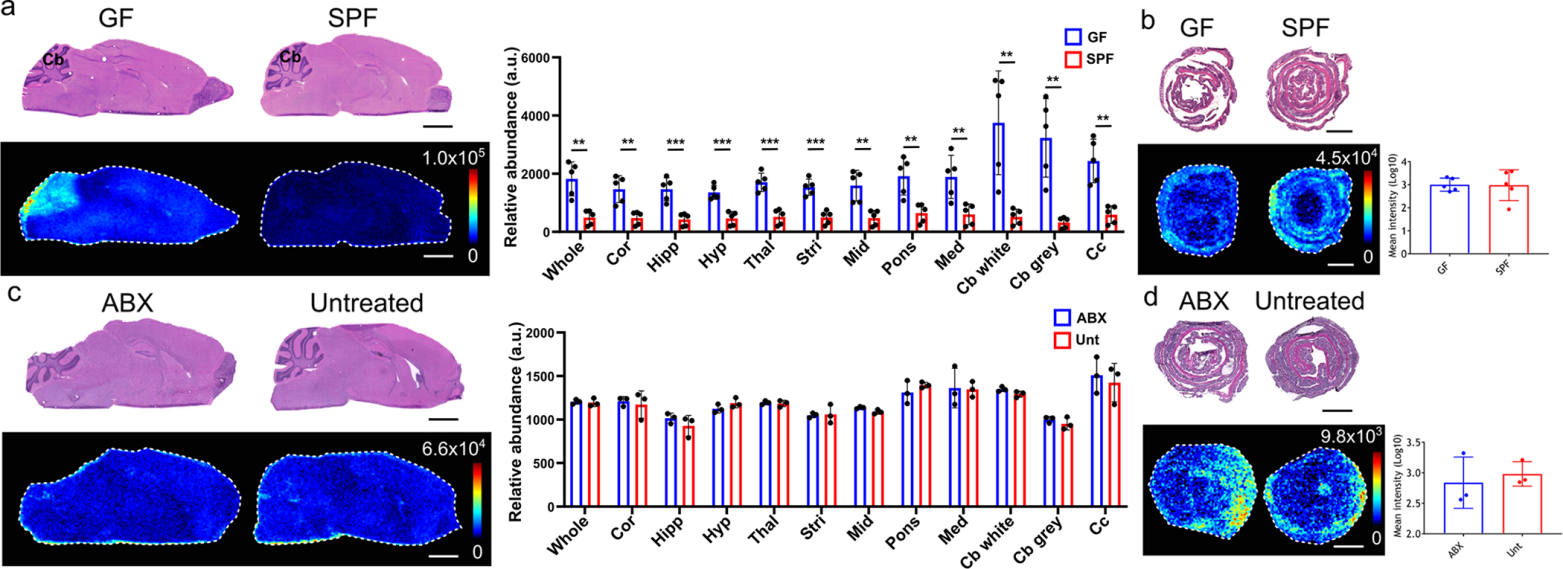
Impact of the gut microbiome on 3-hydroxy-3-methylgularic acid levels in the gut and brain. DESI-MS images of HMG ([M – H]^−^*m*/*z* 161.045) in the (a) GF and SPF mouse brains (*N* = 5), (b) GF and SPF mice colons (*N* = 5), (c) ABX-treated and Unt mouse brain (*N* = 3), and (d) ABX-treated and Unt colons (*N* = 3). H&E stained sections are shown, which are the same tissue sections that underwent DESI-MSI analysis. The bar plots show the average HMG abundance (ion intensity) across the whole tissue sections in the different groups tested. The bar plots for the brain also show relative abundance of neurotransmitters in multiple brain regions the cortex (cor), hippocampus (hipp), hypothalamus (hyp), thalamus (thal), striatum (stri), midbrain (mid), pons, medulla (med), white matter of the cerebellum (Cb white), gray matter of the cerebellum (Cb gray), and the corpus callosum (cc). Annotation in (a) Cb, cerebellum. Statistical analysis was performed using an unpaired *t* test. For individual brain regions statistical analysis was performed using an unpaired *t* test. The *p* values were corrected for multiple testing by False Discovery Rate (FDR), two-stage step-up method. The desired FDR was set to 1%. The asterisks on the bar plot show significance with a *p* value of 0.01 (**), 0.001 (***), or ≤0.001 (****). Brain regions without asterisks were not significantly different in levels of metabolite. Error bars represent standard deviation *N* = 5. Scale bars = 2 mm. Ion images show target *m*/*z* ± 0.005 Da.

The molecule detected at *m*/*z* 161.0446 in negative-ionization mode was found at significantly higher levels (*P* ≤ 0.0001) across the whole brain section of GF compared to SPF mice, with levels significantly higher in the gray and white matter of the cerebellum (10-fold and 7-fold, respectively) (Figure 2a). The change in the brain was not reflected in the gut as levels in the colon did not significantly change between GF and SPF control mice (Figure 2b). The metabolite was identified as [M – H]^−^ of 3-hydroxy-3-methylglutaric acid (3-HMG) by searching the Human Metabolome Database^48,49^ and then confirmed using tandem mass spectrometry (MS/MS) analysis and comparison to an HMG standard (Supplementary Figure 5). No significant difference in abundance of HMG was found in brains and colon after ABX treatment compared to controls (Figure 2c,d).

Quantification analysis of HMG revealed an average concentration of 0.56 μg/g of tissue in the GF brain compared to <0.01 μg/g of tissue in the SPF brain (Supplementary Figure 6). The concentration of HMG was particularly high in the cerebellum in the GF mouse brain at a concentration of 5.02 μg/g of tissue compared to <0.01 μg/g of tissue in the SPF mouse brains.

### Vitamin B5 (*m*/*z* 218.1030) Levels Are Decreased in the Brain of GF Mice

**Figure 3:**
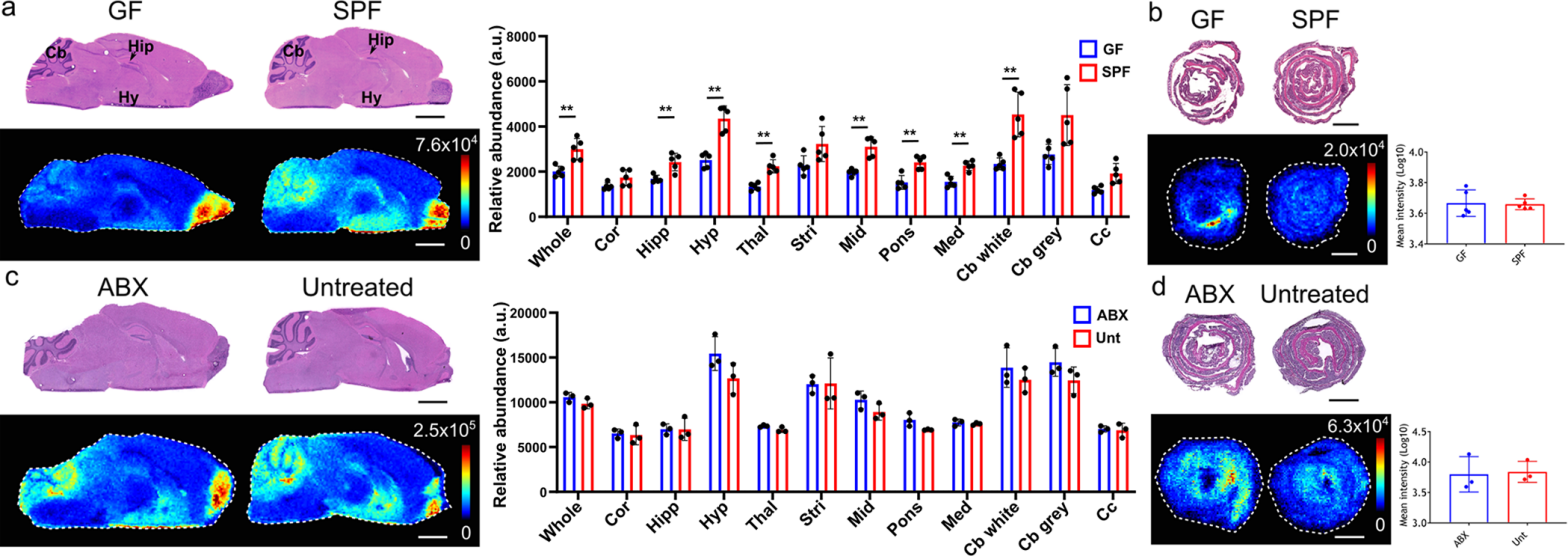
Effects of the gut microbiome on pantothenic acid (B5) levels in the gut and brain. DESI-MS images of B5 ([M – H]^−^*m*/*z* 218.103) in the (a) GF and SPF mouse brains (*N* = 5), (b) GF and SPF mice colons (*N* = 5), (c) ABX-treated and Unt mouse brain (*N* = 3), and (d) ABX treated and Unt colons (*N* = 3). H&E stained sections are shown, which are the same tissue sections that underwent DESI-MSI analysis. The bar plots show the average B5 abundance (ion intensity) across the whole tissue sections in the different groups tested. The bar plots for the brain also show relative abundance of neurotransmitters in multiple brain regions the cortex (cor), hippocampus (hipp), hypothalamus (hyp), thalamus (thal), striatum (stri), midbrain (mid), pons, medulla (med), white matter of the cerebellum (Cb white), gray matter of the cerebellum (Cb gray), and the corpus callosum (cc). Annotation in (a) Cb, cerebellum; Hip, hippocampus; Hy, hypothalamus. Statistical analysis was performed using a paired *t* test. For individual brain regions statistical analysis was performed using an unpaired *t* test. The *p* values were corrected for multiple testing by False Discovery Rate (FDR), two-stage step-up method. The desired FDR was set to 1%. The asterisks on the bar plot show significance with a *p* value of 0.01 (**), 0.001 (***), or ≤0.001 (****). Brain regions without asterisks were not significantly different in levels of metabolite. Error bars represent standard deviation *N* = 5. Scale bars = 2 mm. Ion images show target *m*/*z* ± 0.005 Da.

The second of the unknown molecules had an *m*/*z* of 218.1030 in negative-ionization mode and was found to be significantly lower in the brain of GF mice compared to SPF controls (Figure 3a; *P* ≤ 0.05). This difference was most obvious in the gray and white matter of the cerebellum (1.6-fold and 1.9-fold respectively), the hypothalamus (1.7-fold), and the thalamus (1.7-fold). This molecule was unchanged between GF and SPF colons. The metabolite was identified as [M – H]^−^ of pantothenic acid, also known as vitamin B5, through a database search and subsequent confirmatory MS/MS analysis (Supplementary Figure 7).

No difference was found in the levels of vitamin B5 in the brain or colon in ABX-treated mice compared to untreated control mice (Figure 3a,b). Quantification analysis of vitamin B5 revealed an average concentration of 0.25 μg/g of tissue in the GF brain compared to 0.36 μg/g of tissue in the SPF brain (Supplementary Figure 8).

## Discussion

Studying the MGB axis has significant potential to help us understand and potentially treat, via the microbiome, certain neurological conditions. However, in order to achieve this goal, a greater understanding of MGB communication is required. The techniques applied to date to discover mediators of communication across the MGB axis have typically been targeted toward specific neurotransmitters, metabolites, or regions of the brain.^14,19,26^ Such approaches are often dependent on analyte extraction from brain tissue prior to analysis, meaning data regarding spatial localization within the brain was limited. Here, we applied MSI to study the metabolic processes along the MGB axis, permitting imaging of multiple neurotransmitters in the presence and absence of a microbiome while untargeted MSI also allowed the discovery of novel metabolites involved in MGB axis communication.

Serotonin, tryptophan, dopamine, tyrosine, 3-methoxytyramine, GABA, and glutamate were first imaged to detect changes across the brain and gut of GF animals. No changes were detected when imaging levels of each across whole brain sections while in the intestine only tyrosine and glutamate were increased, and serotonin decreased in GF animals compared to SPF controls. Significantly lower levels of serotonin have previously been detected in the colon of GF mice along with increased levels of the serotonin precursor tryptophan.^32^ No difference in serotonin or tryptophan levels were detected in the brain of GF compared to SPF mice in contrast to previous GF work focused on these neurotransmitters.^14,50^ One week antibiotic (ABX) treated mice were also tested for changes in levels of serotonin and tryptophan in the gut and brain compared to untreated controls. Tryptophan abundance increased in both the colon and the brain of ABX-treated mice compared to untreated controls, but serotonin levels remained unchanged. Tryptophan levels were seen to increase significantly in every brain region imaged in ABX mice. This data mirrors that previously obtained with a similar model, where ABX treatment increased systemic tryptophan while serotonin levels remained unaffected.^19,34^

Data comparison between microbiome studies is complex with prior studies employing rat or mouse models as well as different experimental tools while also highlighting that differences in these molecules can be sex-specific, complicating comparison with our own data. While microbiome changes within mouse colonies as well as between mouse strains are well documented, phenotypic comparisons between GF rodent models which lack a microbiome have also been limited.^51−53^

Dopamine and its precursor tyrosine were also affected by microbiota disruption or absence. Increased tyrosine and dopamine levels were detected in the colon in ABX-treated mice, while GF mice had increased intestinal tyrosine levels compared to controls. However, no difference was found in the abundance of either of these molecules in the brains of GF or ABX mice, with no single specific brain region determined to have significantly changed levels of either molecule.

Previous work found a decrease in the level of dopamine in the guts of GF mice, while another found no difference in the levels of dopamine in the colon after ABX treatment, although antibiotic treatments varied by constitution and duration between ours and previous studies.^16,28^ As tyrosine can be metabolized by certain bacterial species, it is possible that, in addition to causing increased tyrosine levels, reduction in these groups could lead to higher production of dopamine.^54^ Conversely, the release of a biologically active free form of dopamine in the gut, via bacterial β-glucuronidase mediated breakdown of a conjugated form of dopamine, could also play a significant role.^28^ Additionally, although there are no differences in the levels of dopamine in the brain, increased levels in the colon could have localized effects. There are dopamine receptors present in the intestine and dopamine, in a similar manner to serotonin, that have been shown to increase water absorption from the gut and regulation of muscle contraction.^28,55,56^

The neurotransmitter, and GABA precursor, glutamate was increased in the colon of GF mice compared to controls. Glutamate and GABA are the main excitatory and inhibitory neurotransmitters of the central nervous system, respectively.^57^ The increase in glutamate levels in GF mice was surprising given the number of bacteria known to produce glutamate in the intestine.^58^ This build up of glutamate is not due to reduced conversion to GABA as we detected no corresponding difference in GABA levels.^59^ No increase in glutamate in the GF brain was detected in these GF mice with high intestinal glutamate levels, but glutamate is primarily metabolized in the splanchnic area and little enters into circulation from the gastrointestinal tract, instead exerting its significant localized effects on the gut including through stimulation of the vagus nerve.^60,61^

Untargeted MSI indicated that four metabolites were significantly altered in the brain, vitamin B5 or pantothenic acid (*m*/*z* 218.1030), 3-hydroxy-3-methylglutaric acid (3-HMG; *m*/*z* 161.0446), and *m*/*z* 160.133. The latter *m*/*z* was determined to be a mixture of two metabolites which are discussed in detail elsewhere.^47^ Vitamin B5 was significantly decreased in GF mice compared to SPF, whereas 3-HMG was increased. Further analysis of vitamin B5 and HMG determined that they were not significantly altered in ABX-treated mice. Vitamin B5 is a precursor for coenzyme A, which is a critically important molecule in many metabolic pathways including neurotransmitter biosynthesis, the TCA cycle, and metabolism of fatty acids, protein, RNA, and histones.^62,63^ Previously, it was thought that only small amounts of vitamin B5 could be produced by the microbiota, but recent work identified a previously uncharacterized group of *Clostridia* that harbor genes for pantothenic acid biosynthesis.^64,65^ It has also recently been determined that levels of vitamin B5 producing bacteria change according to gestational age in preterm infants.^65^ Neurological symptoms of vitamin B5 deficiency were determined in early studies by inducing deficiency in human subjects, which resulted in defects in neuromuscular function and deterioration of mood.^66^ Vitamin B5 deficiency is observed in the human brain across a number of neurodegenerative conditions including Parkinson’s disease (PD), Alzheimer’s disease (AD) and Huntington’s disease (HD).^63,67,68^ The lower levels in the GF mouse brain were particularly apparent in the cerebellum, hippocampus, and hypothalamus regions, and these decreased levels mirrored those detected in the cerebellum of HD, AD, and PD patients and hippocampus of HD and AD patients.^63,67,68^ Despite the potential for a microbial origin for vitamin B5, no change was found in the levels of vitamin B5 in the colons of GF mice making it unclear what role, if any, the microbiota may play in the significant decrease in vitamin B5 levels in the brain. Vitamin B5 crosses into the brain through the BBB via a saturable transporter and levels are maintained in the brain at around 50 times the concentration found in the plasma, meaning that any reduction in microbiota-derived vitamin B5 levels may require more prolonged antibiotic treatment before effects are seen.^10,69,70^

HMG, a metabolite involved in leucine degradation and ketogenesis, was present at higher levels across the brains of GF mice compared to normal brains, but particularly in the cerebellum. Individuals with the genetic disorder 3-hydroxy-3-methylglutaric aciduria, which leads to a build up of metabolites including HMG, suffer from neurological symptoms including seizures and abnormalities in the brain.^71^ It has been proposed that this accumulation of metabolites is directly affecting the brain with HMG inducing oxidative damage.^71−74^ The particularly high levels of HMG in the cerebellum, a brain region involved in motor control, suggests this region would be most affected in the GF mice. Histopathology analysis of a cat brain with HMG accumulation, due to 3-hydroxy-3-methylglutaric aciduria, showed cerebellum changes along with changes in gait.^75^ While gait changes in GF mice have not been reported, increased motor activity compared to control mice has been reported.^12^ The presence of HMG in the serum has been associated with increased gut permeability in children with environmental enteric dysfunction.^76^ Studies have shown that bacteria play a role in maintaining intestinal barrier function; therefore, the increased intestinal permeability seen in the GF intestine could be leading to higher levels of HMG in circulation compared to normal mice.^77^ Furthermore, intraperitoneal injections of HMG into rats leads to high levels accumulating in the brain of 7 day old rats but not in 30 day old rats in what was speculated to be a BBB permeability related effect.^71^ It has been demonstrated that BBB tight junction proteins such as claudin-5, levels of which are decreased in the absence of a gut microbiota, are essential for BBB function and their absence increases permeability to small molecules.^10,78^ Therefore, given the intestinal permeability defects and the size selective permeability in the BBB in GF mice, HMG, and other small metabolites, are likely entering the circulation and subsequently the brain at higher levels.^10^ Furthermore, no significant difference was found in the levels of HMG in the brain of ABX treated mice where such BBB defects have not been described.

## Conclusions

This study has highlighted the capabilities and potential of MSI to enhance investigation of the MGB axis through the detection and discovery of molecules involved in MGB communication. Here, we show that, despite significant changes in gut microbiota, neurotransmitters are not significantly changed in the brain. As the significance of the MGB axis is still being realized, MSI offers a unique opportunity to understand the complexity of these interactions by identifying both the known and unknown mediators of host–microbe communication.

## Supporting Information Available

MALDI-MSI analysis; 3-hydroxy-3-methylglutaric acid (HMG) and pantothenic acid (vitamin B5) quantitation; Figure S1, brain annotations; Figure S2, pantothenic acid detected ions; Figure S3, effects of the gut microbiome on neurotransmitters, neurotransmitter precursors and neurotransmitter metabolites in the murine brain and gut; Figure S4, effects of the gut microbiome on neurotransmitters, neurotransmitter precursors and neurotransmitter metabolites in the murine brain and gut (post-ABX treatment); Figure S5, identification of metabolite at *m*/*z* 161.0446; Figure S6, absolute quantification of HMG in GF and SPF brain; Figure S7, identification of the metabolite at *m*/*z* 218.102; Figure S8, absolute quantification of vitamin B5 in GF and SPF brain (PDF)

## Notes

**Ethics Approval and Consent to Participate.** Approval for animal procedures was given prior to their initiation by internal University of Manchester and University of Glasgow ethics committees and by the U.K. Home Office under licenses 70/7815, PPL40/4500, P64BCA712 and P78DD6240.

## Notes

**Availability of Data and Material.** The data sets acquired and/or analyzed during the current study are available from the corresponding author on reasonable request.
