# Supplementary material for: Mapping the Influence of the Gut Microbiota on Small Molecules across the Microbiome Gut Brain Axis
Source: J Am Soc Mass Spectrom. 2022 Mar 9;33(4):649–59. doi: 10.1021/jasms.1c00298 (PMC9047441; doi:10.1021/jasms.1c00298)
Supplement: Supplementary file 1 — js1c00298_si_001.pdf [file js1c00298_si_001.pdf]

Mapping the influence of the gut microbiota on small molecules across the microbiome gut brain axis

Heather Hulme[1], Lynsey M. Meikle[1], Nicole Strittmatter[2], John Swales[2], Gregory Hamm[2], Sheila L. Brown[3], Simon Milling[1], Andrew S. MacDonald[3], Richard J.A. Goodwin[2], Richard Burchmore[1], Daniel M. Wall[1,*]

[1]Institute of Infection, Immunity and Inflammation, College of Medical, Veterinary and Life Sciences, Sir Graeme Davies Building, University of Glasgow, Glasgow G12 8TA, United Kingdom

[2]Imaging and Data Analytics, Clinical Pharmacology and Safety Sciences, Biopharmaceuticals R&D, AstraZeneca, Cambridge, CB4 0WG, UK

[3]Lydia Becker Institute of Immunology and Inflammation, Faculty of Biology, Medicine and Health, Manchester Academic Health Science Centre, University of Manchester, Manchester M13 9NT, UK

Corresponding author email: Donal.Wall@glasgow.ac.uk

**Corresponding author address:*

Dr. Daniel (Dónal) M. Wall

Institute of Infection, Immunity and Inflammation

College of Medical, Veterinary and Life Sciences

Sir Graeme Davies Building

University of Glasgow

120 University Place

Glasgow G12 8TA

Supporting information for publication

Supplementary methods

MALDI-MSI analysis

For quantification analysis of HMG, MALDI-MSI analysis was performed using the following parameters: 9-aminoacridine matrix was used at a concentration of 10 mg/mL in 80% methanol 20% water and sonicated for 20 min. The matrix was applied using an automated matrix applicator (HTX technologies, Chapel Hill, NC, US) for 3 passes, using a nozzle temperature of 75°C, gas pressure of 6 psi, velocity of 1120 mm/min and flow rate of 80 µL/min.
MALDI-MSI was carried out in negative ion mode on a Rapiflex MALDI-TOF instrument (Bruker Daltonics, Bremen, Germany) with a 10 kHz smartbeam laser. A spatial resolution of 50 µm was achieved using a single 5 µm laser beam mode, with 600 laser shot over an area of 50x50 µm. Results were analysed using Fleximaging v5 software and the data was normalized to total ion count.

3-hydroxy-3-methylglutaric acid (HMG) and pantothenic acid (vitamin B5) quantitation

Labelled standards for 3-hydroxy-3-methyl-d3-pentanedioic acid and vitamin B5 (*di-β-alanine*-$^{13}C_6$,$^{15}N_2$) calcium salt (Sigma-Aldrich, Poole, Dorset, UK) were spotted onto a brain section using a Preddator reagent dispenser (Redd & Whyte, London, UK). Brain sections with the spotted standards were imaged alongside the experimental brain sections from SPF and GF mice using DESI-MSI for vitamin B5 and MALDI-MSI for HMG. The standards were then used to generate standard curves for mean intensity against concentration for each standard spot. These values were used to calculate the concentration of the endogenous molecule across the experimental brain sections considering the thickness and area of tissue section analysed.

Supplementary figures

Figure S1

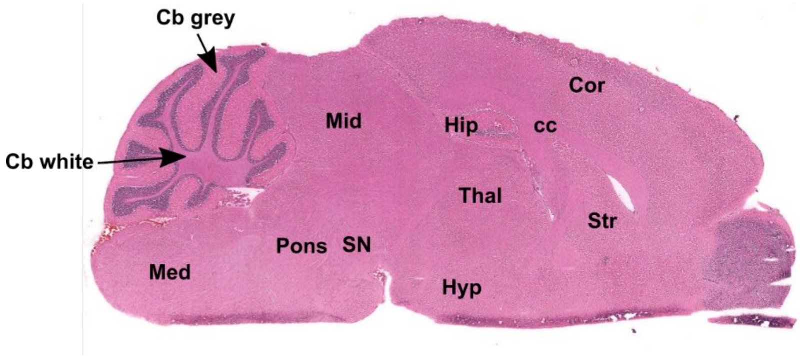

H and E image showing annotated brain regions, which were used for MSI analysis of relative abundance of neurotransmitters and metabolites. Annotation was performed according to Allen mouse brain atlas (43, 44).

Figure S2

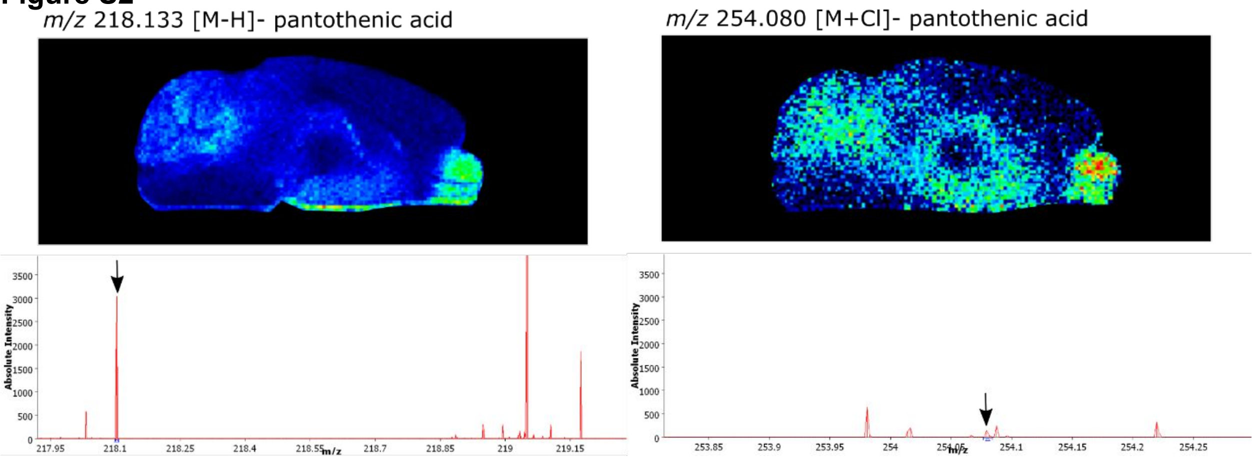

Ion images and mean mass spectra from across the whole tissue section of pantothenic acid [M-H]- and [M+Cl]-. [M-H]- at *m/z* 218.133 was more abundant, therefore was used for quantitation analysis for higher accuracy.

Figure S3

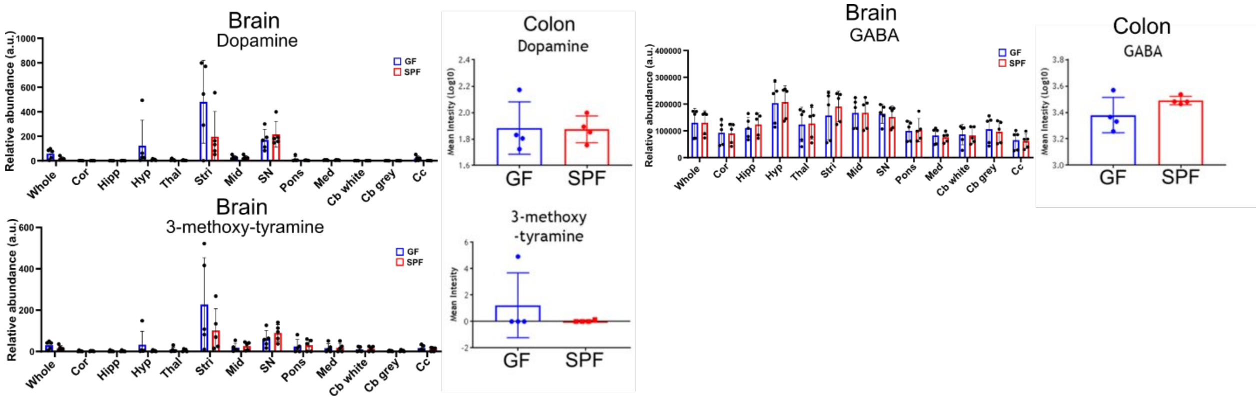

Effects of the gut microbiome on neurotransmitters, neurotransmitter precursors and neurotransmitter metabolites in the murine brain and gut. Bar plots showing the average metabolite abundances in the brain and colon of germ-free mice (GF) compared to specific-pathogen-free mice (SPF). The brain bar plots show relative abundance from multiple brain regions the cortex (cor), hippocampus (hipp), hypothalamus (hyp), thalamus (thal), striatum (stri), midbrain (mid), pons, medulla (med), white matter of the cerebellum (Cb white), grey matter of the cerebellum (Cb grey) and the corpus callosum (cc). Error bars represent standard deviation. Brain N=5, colons N=4.

Figure S4

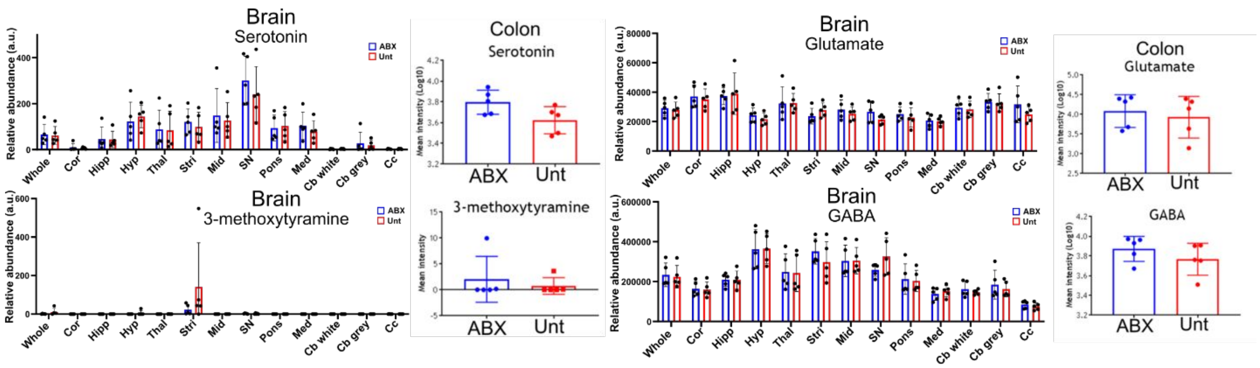

Effects of the gut microbiome on neurotransmitters, neurotransmitter precursors and neurotransmitter metabolites in the murine brain and gut. Bar plots showing the average metabolite abundances in the brain and colon of one week antibiotic treated mice (ABX) compared to untreated control mice (Unt). The brain bar plots show relative abundance from multiple brain regions the cortex (cor), hippocampus (hipp), hypothalamus (hyp), thalamus (thal), striatum (stri), midbrain (mid), pons, medulla (med), white matter of the cerebellum (Cb white), grey matter of the cerebellum (Cb grey) and the corpus callosum (cc). Error bars represent standard deviation. N=5.

Figure S5

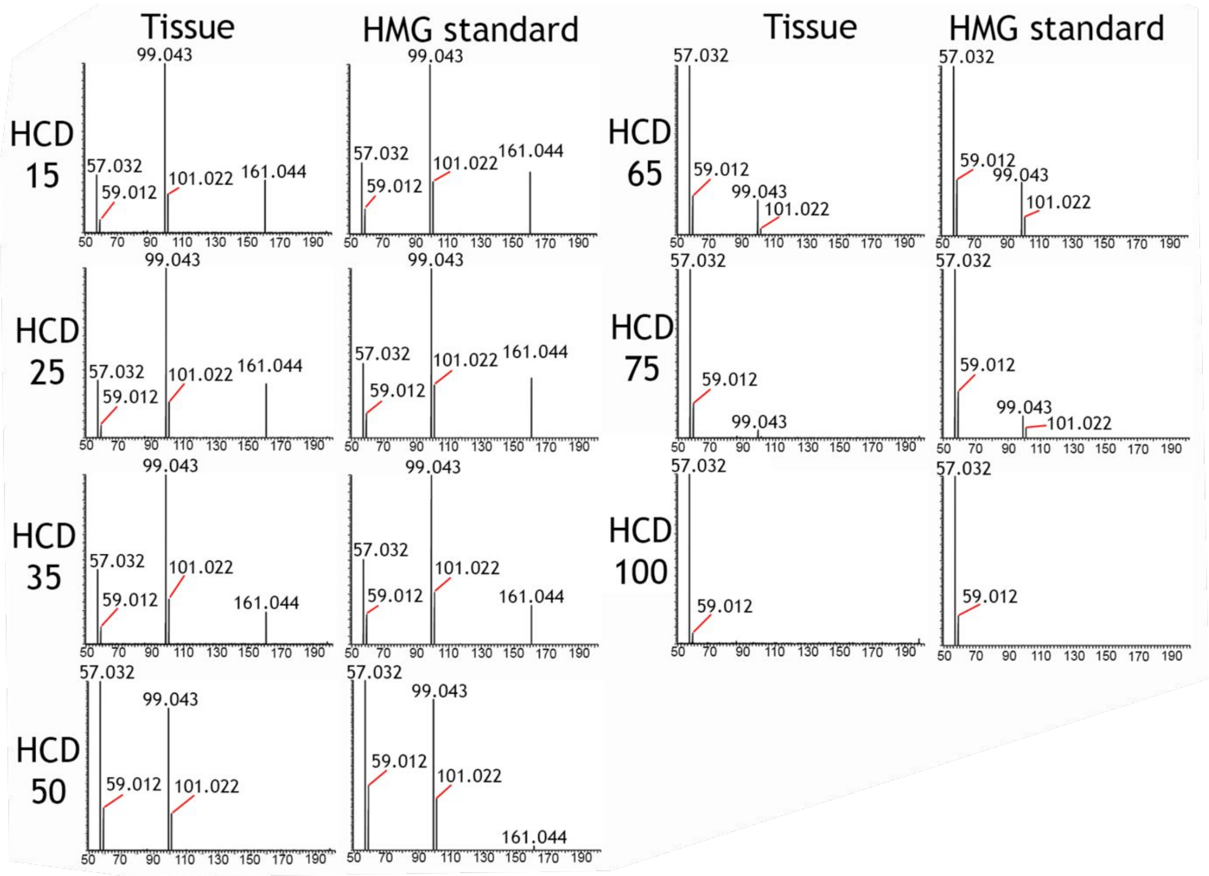

Identification of metabolite at *m/z* 161.0446. Product ion spectra from MS/MS analysis of the metabolite at m/z 161.0446 compared to product ion spectra from MS/MS analysis of 3-hydroxy-3-methylglutaric acid standard. The spectra show the m/z range across the x-axis and arbitrary unit on the y-axis. The MS/MS analysis was performed at different high-energy collision dissociation voltages (HCD 15-100).

Figure S6

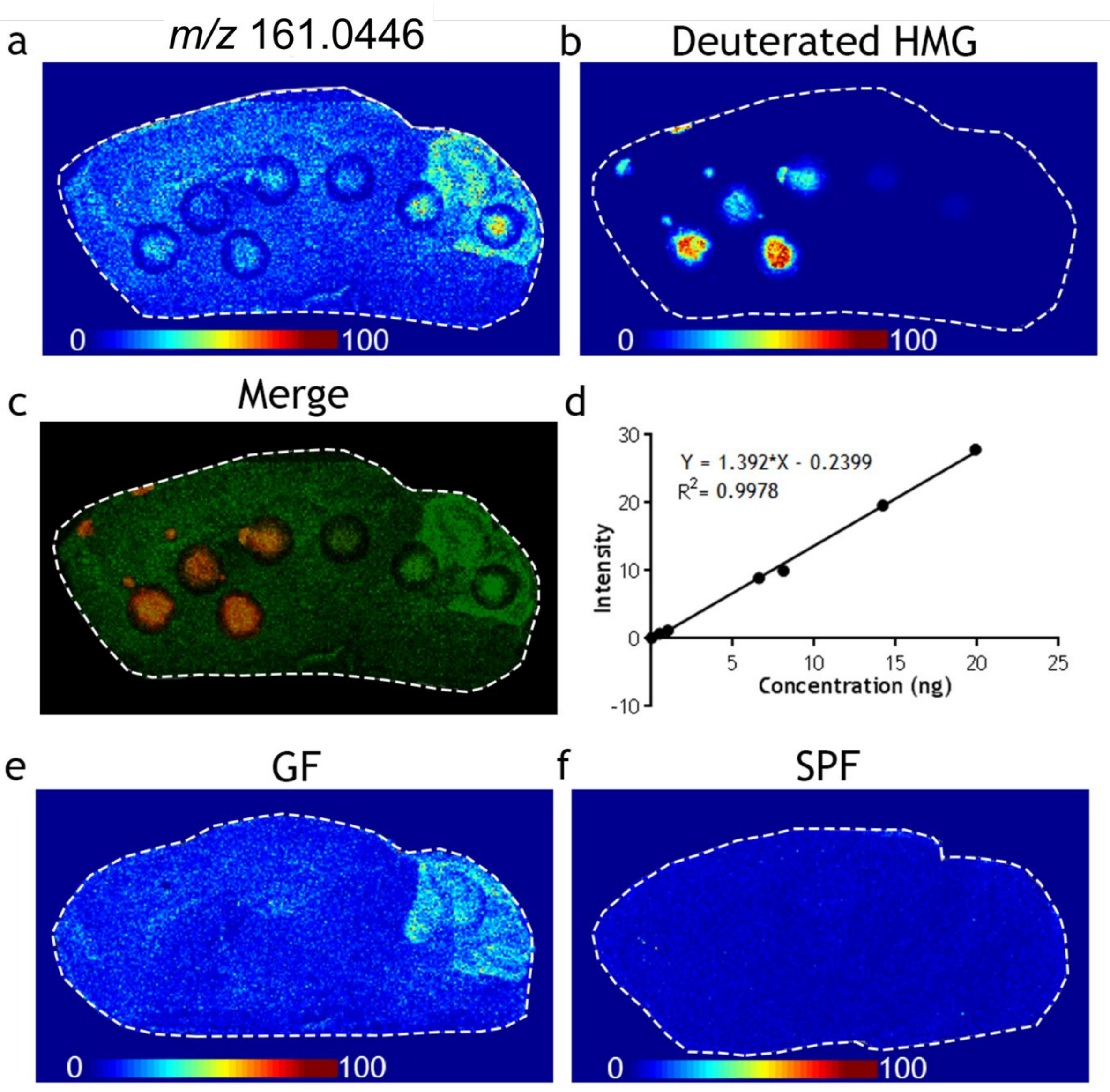

g

	GF	SPF
Whole brain	0.56 μg/g	<0.01μg/g
Cerebellum	5.02 μg/g	<0.01μg/g

Absolute quantification of HMG in GF and SPF brain. (a) MALDI-MSI ion image of HMG ([M-H]- *m/z* 161.044) in the GF brain, which has been spotted with various concentrations of deuterated HMG standard, shown in (b). (c) Overlay of endogenous HMG and deuterated HMG standard. (d) Calibration curve obtained from the various concentrations of deuterated HMG standard spotted on the brain section. (e-g) Comparison of the HMG MALDI-MSI ion images in the GF and SPF mouse brain and absolute quantification values across the whole brain and the cerebellum.

Figure S7

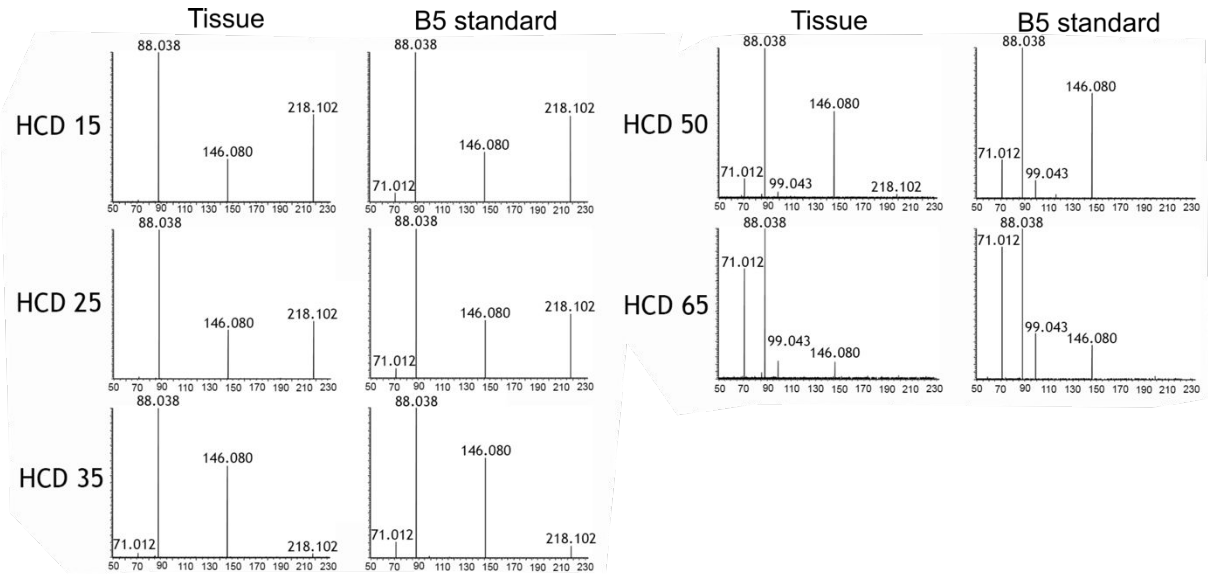

Identification of the metabolite at *m/z* 218.102. Product ion spectra from MS/MS analysis of the metabolite at m/z 218.102 compared to product ion spectra from MS/MS analysis of vitamin B5 standard. The spectra show the m/z range across the x-axis and arbitrary unit on the y-axis. The MS/MS analysis was performed at different high-energy collision dissociation voltages (HCD 15-65).

Figure S8

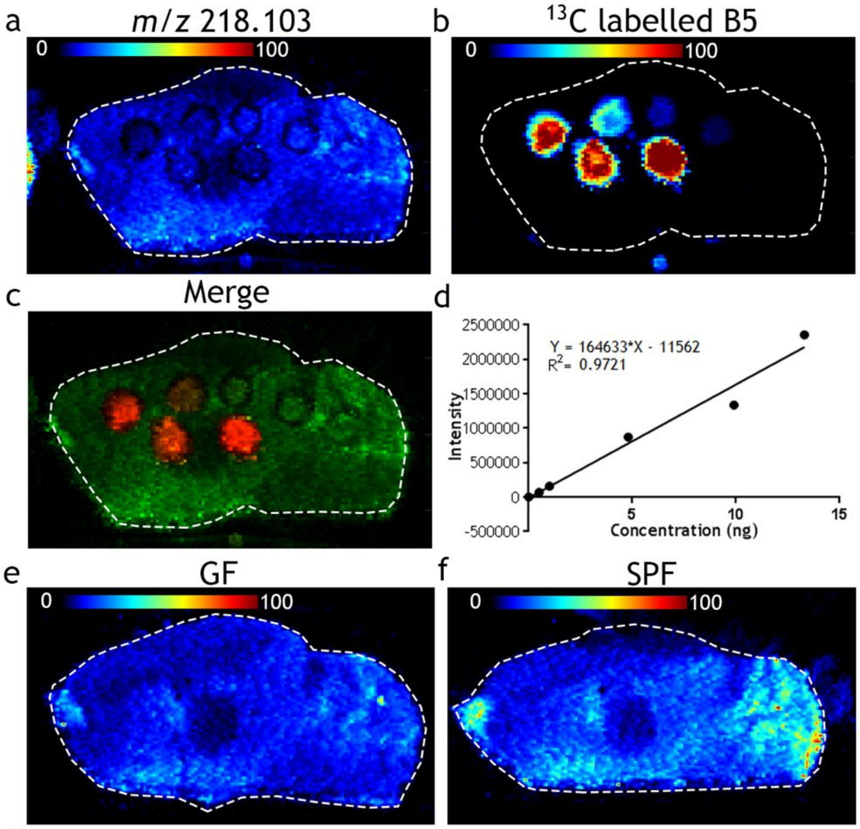

g

	GF	SPF
Whole brain	0.2467 μg/g	0.3574 μg/g

Absolute quantification of vitamin B5 in GF and SPF brain. (a) DESI-MSI ion image of B5 (*m/z* 218.103) in the GF brain, which has been spotted with various concentrations of ^{13}C labelled B5 standard, shown in (b). (c) Overlay of endogenous HMG and ^{13}C labelled B5 standard. (d) Calibration curve obtained from the various concentrations of deuterated ^{13}C labelled B5 standard spotted on the brain section. (e-g) Comparison of the B5 DESI-MSI ion images in the GF and SPF mouse brain and absolute quantification values across the whole brain and the cerebellum.
